# Occupational lifting predicts hospital admission due to low back pain in a cohort of airport baggage handlers

**DOI:** 10.1007/s00420-019-01470-z

**Published:** 2019-08-26

**Authors:** Charlotte Brauer, Sigurd Mikkelsen, Ellen Bøtker Pedersen, Karina Lauenborg Møller, Erik Bruun Simonsen, Henrik Koblauch, Tine Alkjær, Karin Helweg-Larsen, Lau Caspar Thygesen

**Affiliations:** 1 Department of Occupational and Environmental Medicine, Bispebjerg University Hospital, Bispebjerg Bakke 23, 2400 Copenhagen, Denmark; 2 National Institute of Public Health, University of Southern Denmark, Studiestræde 6, 1455 Copenhagen, Denmark; 3 Department of Neuroscience and Pharmacology, Panum Institute, University of Copenhagen, Blegdamsvej 3B, 2200 Copenhagen, Denmark; 4 Department of Biomedical Sciences, University of Copenhagen, Blegdamsvej 3B, 2200 Copenhagen, Denmark; 5 The Department of Physical and Occupational Therapy, Bispebjerg University Hospital, Bispebjerg Bakke 23, 2400 Copenhagen, Denmark

**Keywords:** Dose–response relationship, Longitudinal study, Manual material handling, Musculoskeletal disorders, Low back disorders

## Abstract

**Purpose:**

To examine if occupational lifting assessed as cumulative years as a baggage handler is associated with first-time hospital diagnosis or treatment for low back disorders.

**Methods:**

This study is based on the Copenhagen Airport Cohort consisting of male baggage handlers performing heavy lifting every day and a reference group of unskilled men from the greater Copenhagen area during the period 1990–2012. We followed the cohort in the National Patient Register and Civil Registration System to obtain information on diagnoses, surgery, mortality, and migration. The outcomes were first-time hospital diagnosis or surgery for (1) lumbar disc herniation or (2) low back pain (LBP).

**Results:**

Baggage handlers (*N* = 3473) had a higher incidence rate of LBP, but not of lumbar disc herniation, compared to the reference group (*N* = 65,702). Baggage handlers with longer employment had a higher incidence of LBP compared to baggage handlers with shorter employment. The linear association of cumulative years as a baggage handler on LBP was significantly increased with an incidence rate ratio of 1.16 (95% CI 1.07–1.25) for a 5-year increase of employment as baggage handler.

**Conclusions:**

In this large cohort study, we found an increased incidence of LBP among baggage handlers compared to the reference group with indications of a dose–response relationship between years of employment and the outcome. For baggage handlers working on the apron, the incidence was particularly increased before introduction of technical lifting equipment, suggesting that preventive measures to reduce cumulative work load may have a positive effect.

## Introduction

Low back pain (LBP) is a common problem affecting most adults at some point in life. LBP is often recurrent and may have substantial consequences in terms of work absence or disability pension (Airaksinen et al. 2006; Hoy et al. 2010; Waddell and Burton 2001). LBP covers different diagnoses where the most common diagnoses are unspecified disorders of the back and intervertebral disc disorders (Martin et al. 2008). Individual risk factors for LBP are a previous history of LBP, age, obesity, smoking, low socioeconomic status, depressive mood, and somatization tendency (De Beeck and Hermans 2000; Pincus et al. 2002; Plouvier et al. 2009; Shiri et al. 2019; Wahlstrom et al. 2012). Occupational factors related to the psychosocial as well as the physical work environment have also been identified as potential risk factors for LBP. There are indications of an effect of low social support in the workplace and low job satisfaction on LBP (Hoogendoorn et al. 2000; Kaila-Kangas et al. 2004). Heavy physical work, lifting, and working in awkward postures have been identified as potential work-related risk factors for LBP (Bernard 1997; Coenen et al. 2014; De Beeck and Hermans 2000; Gallagher 2005; Griffith et al. 2012; Hansson 2001; Harms-Ringdahl et al. 2014; Heneweer et al. 2011; Lotters et al. 2003). A few studies have found an association between lumbar disc herniation and whole-body vibrations and lifting/carrying (Seidler et al. 2003; Wahlstrom et al. 2018). It is still unclear, however, whether heavy physical work and manual material handling are associated with lumbar disc herniation. A genetic predisposition seems to be the most important risk factor for lumbar disc herniation (Battie et al. 2009; Martirosyan et al. 2016).

However, most studies examining physical work-related factors and low back disorders have used self-reported information on exposure or outcome and are cross-sectional with methodological weaknesses (Barrero et al. 2009; Lenderink et al. 2012; Stock et al. 2005). In the longitudinal studies, the follow-up time is generally short (Coenen et al. 2014) which may be a problem, as low back disorders may take years to develop. Hence, there is a need for longitudinal studies with long follow-up and objective and independent information on occupational lifting and LBP and lumbar disc herniation.

It is well documented that most airport baggage handlers are exposed to heavy lifting in their daily work and that they report a high prevalence of pain, especially in the low back, knees, and shoulders (Bergsten et al. 2015; Bern et al. 2013; Stalhammar et al. 1986; Tafazzol et al. 2016; Undeutsch et al. 1982). Biomechanical studies have shown that the lumbar muscle activity is high among baggage handlers in all tasks and that the lumbar compression forces on L4–L5 disc exceed the recommended limits for compression during lifting (Koblauch 2016; Tafazzol et al. 2016). The work environment of airport baggage handlers is internationally relevant and will still be relevant in the future with the increasing air traffic (Statista—Statistics and Market Data about transport and Logistics 2019; The International Air Transport Association (IATA) 2019).

In 2012, we established The Copenhagen Airport Cohort, a historical cohort consisting of an exposed group of airport baggage handlers performing heavy lifting every day and a reference group of unskilled workers. The cohort has a long follow-up time and objective information on job function and employment as well as information on diagnoses and surgery from Danish national registers (Moller et al. 2017). One purpose of establishing the cohort was to assess associations between manual material handling in awkward postures and the incidence of low back disorders and other musculoskeletal disorders. We have found an increased incidence of hospital admissions due to meniscal lesions in the knees and subacromial shoulder disorders among baggage handlers compared to the reference group and the incidence increased with years of employment (Mikkelsen et al. 2016; Thygesen et al. 2016).

The purpose of the present study was to examine if occupational lifting assessed as cumulative years as a baggage handler is a predictor for first-time hospital diagnosis or treatment for LBP and lumbar disc herniation. In addition, we wished to examine whether the incidence of these low back disorders differed between baggage handlers working on the apron and in the baggage sorting area in the terminal and whether the incidence decreased after introduction of technical lifting equipment.

## Study population and methods

### Cohort

The Copenhagen Airport Cohort consists of baggage handlers with employment at Copenhagen Airport (CPH) and a reference group of men with unskilled work and a similar socioeconomic background but with less physical workload during the period 1990–2012 (*n* = 69,175). Only men were included as there were no women working as baggage handlers at CPH. We included both present and former employees who were registered in electronic employment registers as well as trade union membership registers. A good agreement was found between the data sources (Mikkelsen et al. 2016; Moller et al. 2017). For each person, we got information on all employment and union membership periods as well as job title and dates for work periods in specific departments. Hence, we could identify baggage handling work and other work tasks for each calendar year since first entry date. A person was classified as a baggage handler if he had ever worked as such. The baggage handler cohort consisted of baggage handlers from the two largest handling companies at CPH. The reference cohort consisted of men employed in other unskilled occupations at the airport (e.g., security personnel, cleaning, and area maintenance personnel) or in other firms in the Greater Copenhagen area (e.g., drivers, postal workers, garbage collectors, and municipal workers). The construction of the cohort is described in detail by Moller et al. (2017).

All Danish residents have a unique personal identification number, and hence, we could follow the cohort in the National Patient Register, the Civil Registration System, and registers at Statistics Denmark to obtain information on diagnoses, surgery, mortality, migration, retirement, and educational level (Lynge et al. 2011; Pedersen 2011; Thygesen et al. 2011).

In addition, we collected self-reported data by a questionnaire survey in 2012 about lifestyle factors such as body mass index (BMI), leisure-time physical activity, and smoking habits (Bern et al. 2013). The questionnaire was sent to all baggage handlers and a random sample of the reference group. Non-responders were contacted by phone. The questionnaire was sent to 5474 persons in the cohort of whom 3749 responded (68%) with no difference between baggage handlers and reference group.

### Baggage handler work

The baggage handlers either work in the baggage sorting area inside the terminal building or outdoors on the apron where the aircraft are parked at the gates. In the baggage sorting area, they load or unload baggage carts or containers to or from a belt conveyor. On the apron, they work either on the ground or inside the baggage compartment of the aircraft loading or unloading most aircraft manually. During bulk loading and unloading an aircraft, one baggage handler is standing at the belt loader on the ground transferring baggage between baggage carts and the belt, while another baggage handler is inside the baggage compartment where he stacks the baggage. The baggage handler inside the baggage compartment either works in sitting, kneeling, squatting, or stooped postures depending on the height of the compartment and personal preference.

We received detailed information about flights and load from the baggage handling companies. There are seasonal and interpersonal variations, but baggage handlers on average lift or handle approximately 5000 kg in a 9-h shift and a piece of baggage weighs on average 15 kg (Mikkelsen et al. 2016; Moller et al. 2017). The daily baggage handling load for each baggage handler has been rather constant over years since 1990. Technical lifting equipment in the form of baggage lifters with a hook was introduced in the baggage sorting area in 1998, and on the apron, extendable belt loaders were introduced from 2002 to 2004. These belt loaders can extend into the baggage compartment and have a height adjustable section of the conveyor, so the luggage can be pushed or pulled instead of being lifted.

### Exposure

The primary exposure measure was cumulative years as a baggage handler. We computed the percentage of employment as a baggage handler for each year and then cumulated the percentage of employment during follow-up resulting in the time-dependent cumulative years as a baggage handler available for each year. From the employment registers of the handling companies, we also had information on years of employment on the apron and in the baggage sorting area and cumulated these during follow-up. In addition, we included cumulative years in the baggage sorting area and on the apron before and after 1998 and 2004 to evaluate the influence of lifting equipment and extendable belt loaders, respectively.

### Outcome

The primary outcome of the study was first hospitalization with a diagnosis or surgical treatment of low back disorders. A priori we divided the low back disorders into two groups: (1) lumbar disc herniation and (2) unspecified disorders of the back, in the following referred to as LBP. Data on diagnoses and surgery were obtained from the National Patient Register which includes information on contacts to the secondary health care system since 1977 about hospital discharge diagnoses, surgery codes, date of admission, and date of surgery linked to the unique personal identification number (Lynge et al. 2011). Before 1994, all diagnoses were classified according to the International Classification of Diseases version 8 (ICD-8) and afterwards according to ICD-10. From 1995, data were added on outpatients. Surgical procedures were before 1996 coded according to a Danish national classification system and from 1996 according to the NOMESCO classification of Surgical Procedures (Nordic Medico-Statistical Committee 2011).

In the analyses, we included persons with either a diagnosis or a surgical procedure for lumbar disc herniation and persons with a diagnosis of LBP, respectively. We included only primary diagnoses. For lumbar disc herniation, we included diagnoses describing lumbar, thoracolumbar, and lumbosacral intervertebral disc disorders with or without myelopathy or radiculopathy and surgical procedures for excision of lumbar intervertebral disc displacement or discectomy of lumbar spine. For the diagnosis of LBP, we included diagnoses of lumbago with or without sciatica, low back pain, and sprain/strain of lumbar spine. The specific codes for diagnoses and surgical procedures are listed in the Appendix.

### Covariates

All covariates were chosen a priori based on the literature and a directed acyclic graph (Greenland et al. 1999; Shrier and Platt 2008). The following factors were included in the analyses: age, calendar year, highest attained educational level, and year for introduction of lifting equipment. Age was included both continuously and categorically (< 30, 30–44, 45–59, and 60+ years), calendar year was included categorically (1990–1994, 1995–1999, 2000–2004, and 2005–2012), and educational level was included categorically (elementary school, high school, vocational education, and higher education). Baggage lifting equipment and extendable belt loaders were included as binary covariates before and after 1998 and 2004 for baggage handlers working in the baggage sorting area and on the apron, respectively.

As additional information about the comparability of baggage handlers and referents, we reported the prevalence of the following self-reported factors from the questionnaire survey in 2012: BMI, smoking habits, alcohol consumption, leisure-time physical activity, general health, and low back pain. As descriptive measures of the baggage handlers’ work, we reported information collected in the survey among baggage handlers only (proportion of work in the baggage compartment/on the ground on the apron, working postures in the baggage compartment, and use of lifting equipment in the baggage sorting area).

### Statistical analyses

We followed the cohort from start of employment, 1 January 1990 or immigration after employment, whichever came last, and until first diagnosis or surgical procedures for low back disorders, emigration, death or end of follow-up (31 December 2012), whichever came first. We excluded persons who died or emigrated before 1990 or who only had employment after diagnosis of low back disorders. After exclusions, the cohort for the analyses of lumbar disc herniation consisted of 68,436 men (2579 baggage handlers and 65,857 in the reference group), while the cohort for the analyses for LBP consisted of 67,695 men (2539 baggage handlers and 65,156 in the reference group).

The data for persons with LBP as an outcome were censored if they got a diagnosis or surgery for lumbar disc herniation prior to the diagnosis of LBP, whereas no censoring was done for LBP in the analyses of lumbar disc herniation, because disc herniation may develop in a person who already has LBP.

We included status as baggage handler and cumulative years of employment as baggage handler in four predefined models:

1. Baggage handlers compared to the external reference group as a binary group variable.
2. Baggage handler cumulative years as a categorical variable (non-baggage handler, 0.1–2.9 years, 3.0–9.9 years, 10.0–19.9 years and 20.0+ years). We used the least exposed group of baggage handlers (0.1–2.9 years of employment) as the reference group to assess a dose–response relationship among baggage handlers. In this way, the external reference group only serves as an anchor point for comparison with baggage handlers with short exposure and not as a zero-exposed group.
3. Cumulative years as a continuous variable and including the binary group variable (coded ‘1’ for external referents and ‘0’ for baggage handlers). By coding this way, the influence of cumulative years only refers to baggage handler cumulative years.
4. Cumulative years as a restricted cubic spline with knots at 5th, 27.5th, 50th, 72.5th, and 95th percentiles of the distribution of cumulative years using the SAS macro PSPLINET. In this model, we included the same binary group variable, so the effect of cumulative years refers to baggage handler cumulative years. We included a test for non-linearity of the influence of cumulative years on low back disorders using the likelihood ratio test, comparing the model with only the linear term to the model with the cubic spline terms.

For each model, we performed both an unadjusted analysis and an adjusted analysis where we included the confounders mentioned above.

The impact of changes in diagnosis and surgery classification systems in 1994 and 1996, respectively, was examined in a sensitivity analysis for outcomes that occurred after these changes. Furthermore, we examined if the influence of age was different for baggage handlers and the reference group.

We also analyzed the influence of cumulative years in the baggage sorting area and on the apron where we included cumulative years as categorical variables and used the categories 0.1–2.9 years, 3.0–5.9 years and 6.0+ years because of few cases with long duration of employment in these two areas. We stratified the analyses on calendar years before and after introduction of technical lifting equipment (1998 in the baggage sorting area and 2004 on the apron).

Finally, we evaluated whether risk of low back disorders changed after cessation of employment as baggage handler and if such a change depended on cumulative years as baggage handler. The influence of years since cessation was modeled as a restricted cubic spline. We tested an interaction between cumulative years and years since cessation.

Two-sided *p* values of below 0.05 were considered statistically significant. Data were analyzed using Poisson regression with log-transformation of person-years at risk and Cox regression (restricted cubic spline) using SAS version 9.3 (SAS Institute, Cary, North Carolina, USA).

## Results

The total cohort of 69,175 men consisted of 3473 baggage handlers and 65,702 men in the reference group. At the start of follow-up, the baggage handlers were slightly younger than the reference group and a larger proportion of the baggage handlers had a vocational education than the reference group (Table 1). Self-reported data from the survey in 2012 showed similar BMI, smoking habits, alcohol consumption per week, and leisure-time physical activity (Table 1). Most baggage handlers (70%) reported to work equally on the ground and in the baggage compartment when they were on the apron. Kneeling and sitting were the most frequently reported working postures in the baggage compartment. For work in the baggage sorting area, only 36% reported to use the lifting equipment frequently (half of the time or more).

**Table 1:** Characteristics of the Copenhagen Airport Cohort by baggage handlers and referents

	Baggage handlers		Reference group	
	*N*	%	*N*	%
Data from registers^a^				
*N*	3473		65,702	
*Age groups (years)*				
< 30	2148	61.8	30,203	46.0
30–44	1208	34.8	21,357	32.5
45–59	115	3.3	10,258	15.6
60+	2	0.1	3884	5.9
*Educational level*				
Elementary school	1582	45.6	37,231	56.7
High school	446	12.8	8472	12.9
Vocational education	1296	37.3	16,712	25.4
Higher education	149	4.3	3287	5.0
*Marital status*				
Widower	2	0.1	703	1.1
Divorced	159	4.6	5795	8.8
Married	814	23.4	20,382	31.0
Unmarried	2498	71.9	38,822	59.1
Data from survey 2012				
*Respondents*	1786		1963	
*Body mass index (kg/m*^2^*)*				
< 18.5	1	0.1	9	0.5
18.5–24.9	611	34.6	679	35.2
25–29.9	865	49.0	897	46.5
30.0+	288	16.3	346	17.9
*Smoking*				
Never	682	38.5	680	34.8
Past	606	34.2	674	34.5
Current	484	27.3	598	30.6
*Alcohol (units/week)*				
None	435	24.6	502	25.8
1–21	1248	70.7	1318	67.8
> 21	82	4.6	125	6.4
*Leisure*-*time physical activity*				
Sedentary	175	9.9	255	13.2
Low	624	35.4	697	35.9
Medium	711	40.3	707	36.5
High	254	14.4	280	14.4
*General health*				
Excellent/very good	645	36.5	880	45.3
Good	728	41.2	777	40.0
Fair/poor	394	22.3	286	14.7
*Low back pain the past 12* *months*				
Not at all/a little/somewhat	1124	67.9	1396	76.2
Quite a lot/very much	531	32.1	437	23.8

Register-based information and self-reported information from questionnaire survey

^a^Descriptive statistics for the first year during follow-up that a person was a baggage handler, or the first year during follow-up for workers who were never a baggage handler (reference group)

A diagnosis of lumbar disc herniation was less frequent than LBP. There were 2244 incident cases of lumbar disc herniation cases (118 baggage handlers and 2126 referents) and 3143 incident cases of LBP (237 baggage handlers and 2906 referents) (Table 2). Most diagnoses of lumbar disc herniation were unspecific (DM511) or at the L4/L5 or L5/S1 levels (DM511D and DM511E, respectively).

**Table 2:** Incidence rate ratios (IRR) and 95% confidence intervals (95% CI) based on Poisson regression of the association between work as a baggage handler and low back disorders

		Lumbar disc herniation						Low back pain					
		Cases	Person–years	IR	IRR	95% CI	*P* value	Cases	Person–years	IR	IRR	95% CI	*P* value
Model 1	*Binary group variable*												
	Unadjusted												
	Non-baggage handler	2126	973,528	218	1.00	Reference	0.15	2906	941,557	309	1.00	Reference	< 0.0001
	Baggage handler	118	47,228	250	1.14	0.95–1.38		237	44,784	529	1.71	1.50–1.96	
	Adjusted^a^												
	Non-baggage handler				1.00	Reference	0.26				1.00	Reference	< 0.0001
	Baggage handler				0.82	0.58–1.16					1.54	1.25–1.91	
Model 2	*Cumulative years of employment, categorical*												
	Unadjusted												
	Non-baggage handler	2126	973,528	218	1.01	0.72–1.41	0.48	2906	941,557	309	0.79	0.61–1.02	< 0.0001
	0.1–2.9 years	35	16,202	216	1.00	Reference		61	15,653	390	1.00	Reference	
	3.0–9.9 years	41	16,144	254	1.18	0.75–1.85		84	15,420	545	1.40	1.01–1.94	
	10.0–19.9 years	31	10,569	293	1.36	0.84–2.20		64	9802	652	1.68	1.18–2.38	
	20.0+ years	11	4314	255	1.18	0.60–2.32		28	3909	716	1.84	1.17–2.87	
	Adjusted^a^												
	Non-baggage handler				1.30	0.84–2.02	0.80				0.85	0.63–1.15	< 0.0001
	0.1–2.9 years				1.00	Reference					1.00	Reference	
	3.0–9.9 years				1.07	0.68–1.69					1.38	0.99–1.92	
	10.0–19.9 years				1.15	0.71–1.86					1.62	1.14–2.31	
	20.0+ years				1.05	0.53–2.09					1.94	1.23–3.07	
Model 3	*Cumulative years of employment, continuous linear per 5 years*^*b*^												
	Unadjusted^c^				1.04	0.93–1.17	0.49				1.14	1.06–1.23	0.001
	Adjusted^d^				1.01	0.90–1.14	0.86				1.16	1.07–1.25	< 0.0001

The Copenhagen Airport Cohort of 69,175 men (3473 baggage handlers and 65,702 non-baggage handlers)

*IR* incidence rate per 100,000 person-years

^a^Adjusted for age, use of baggage lifting aids, use of extending belt loader, educational level, and calendar year

^b^Effect of a 5-year increase of employment as baggage handler

^c^Not adjusted for potential confounders, but including the binary group variable for baggage handler (yes/no)

^d^Adjusted for the binary group variable for baggage handler (yes/no) in addition to age, year, use of baggage lifting aids, use of extending belt loader, educational level, and calendar year

In general, baggage handlers had a higher incidence of LBP, but not of lumbar disc herniation compared to the external reference group (Table 2, model 1). When the cumulative years of employment were analyzed in categories, baggage handlers with longer employment had a higher incidence of LBP compared to baggage handlers with shorter employment (Table 2, model 2). Baggage handlers with 20 years of employment or more had an incidence rate ratio (IRR) of 1.94 (95% confidence interval (CI) 1.23–3.07) of LBP compared to baggage handlers with less than 3 years of employment (fully adjusted model). The linear association of cumulative years as a baggage handler on LBP was significantly increased with an adjusted IRR of 1.16 (95% CI 1.07–1.25) for a 5-year increase of employment as baggage handler (Table 2, model 3). Cumulative years did not affect the incidence of lumbar disc herniation in any of the models.

**Fig. 1:**
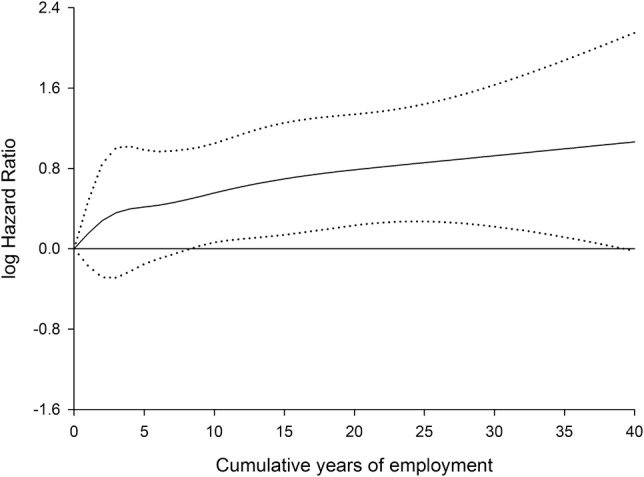
Association between cumulative years as baggage handler and low back pain. Restricted cubic splines with 95% confidence interval. Cox regression model adjusted for baggage handler status (yes/no), age, use of baggage lifting aids, use of extendable belt loader, educational level, and calendar year

Figure 1 shows that the hazard ratio (HR) of LBP increased monotonically with cumulative years as a baggage handler. There was no indication of a cut point where the effect changed, and the spline model was not significantly better than the model with cumulative years included with a linear effect (*P* = 0.72). For lumbar disc herniation the test for non-linearity was also non-significant (*P* = 0.85), but here the line was almost horizontal (not shown). The sensitivity analysis where we restricted to a diagnosis or surgery after 1994 and 1996, respectively, did not alter our results.

Cumulative years as baggage handler and age were highly correlated (Pearson’s correlation coefficient, 0.63), and the effect of cumulative years on LBP (cf. above) may, therefore, be over-adjusted when adjusting for age. The effect of age on LBP showed an adjusted IRR of 1.08 (95% CI 0.95–1.23) among baggage handlers and an adjusted IRR of 0.89 (95% CI 0.86–0.91) in the reference group for a 10-year increase of age. Test for interaction between age and baggage handler status was significant (*P* = 0.011). For lumbar disc herniation, there was no interaction between age and baggage handler status.

The non-baggage handlers had a significantly reduced risk of LBP compared to the baggage handlers in the baggage sorting area as well as on the apron (Table 3). In the baggage sorting area, there was a significant association in the continuous linear model between cumulative years of employment and LBP after 1998 when lifting equipment was introduced, but not before 1998. On the apron, there was an increased incidence of LBP with longer employment before the introduction of technical lifting equipment in 2004 when including employment length continuously (*P* = 0.0003). In the categorical analyses, the baggage handlers with 6 or more years of employment on the apron had a higher incidence of LBP before 2004 with an IRR of 1.54 (95% CI 1.12–2.12) compared to baggage handlers with less than 3 years of employment on the apron, while the IRR was 0.94 (95% CI 0.38–2.36 from 2004 to 2012. We found no effect on lumbar disc herniation for baggage handlers neither in the baggage sorting area nor on the apron.

**Table 3:** Association between lumbar disc herniation and low back pain and cumulative years as baggage handler in the baggage sorting area and at the apron before and after introduction of technical lifting equipment, incidence rate ratio (IRR), and 95% confidence intervals (95% CI)

	Before 1998^a^				1998–2012^a^				Before 2004^a^				2004–2012^a^			
	Cases	IRR	95% CI	*P* value	Cases	IRR	95% CI	*P* value	Cases	IRR	95% CI	*P* value	Cases	IRR	95% CI	*P* value
*Lumbar disc herniation*																
Non-baggage handler	2170	0.93	0.70–1.23	0.36	2181	0.95	0.73–1.25	0.94	2137	0.89	0.65–1.21	0.73	2200	0.89	0.61–1.29	0.68
0.1–2.9 years	50	1.00	Reference		55	1.00	Reference		42	1.00	Reference		28	1.00	Reference	
3.0–5.9 years	22	1.08	0.65–1.79		7	1.14	0.52–2.51		21	0.84	0.50–1.41		12	1.17	0.59–2.30	
6.0+ years	2	0.36	0.09–1.50		1	0.72	0.10–5.23		44	1.01	0.66–1.55		4	1.21	0.42–3.44	
Continuous linear^b,c^		0.85	0.56–1.30	0.45		1.20	0.63–2.32	0.58		1.02	0.86–1.21	0.80		1.50	0.93–2.40	0.09
*Low back pain*																
Non-baggage handler	2980	0.59	0.48–0.71	< 0.0001	3024	0.65	0.53–0.80	< 0.0001	2927	0.80	0.62–1.02	< 0.0001	3069	0.54	0.41–0.71	< 0 .0001
0.1–2.9 years	107	1.00	Reference		100	1.00	Reference		66	1.00	Reference		51	1.00	Reference	
3.0–5.9 years	43	1.04	0.73–1.49		16	1.76	1.04–2.98		57	1.60	1.12–2.28		18	1.08	0.63–1.85	
6.0+ years	13	1.11	0.62–1.97		3	1.56	0.49–4.92		93	1.54	1.12–2.12		5	0.94	0.38–2.36	
Continuous linear^b,c^		1.15	0.91–1.45	0.24		1.72	1.11–2.67	0.02		1.22	1.10–1.36	0.0003		1.33	0.90–1.96	0.16

^a^Adjusted for age, educational level, and calendar year

^b^Also adjusted for baggage handler (yes/no)

^c^Effect of a 5-year increase of employment as baggage handler in the baggage sorting area and at the apron, respectively

Cessation of work as a baggage handler was associated with a decreasing trend in the risk of LBP with time since cessation (HR = 0.72, 95% CI 0.54–0.95, *P* = 0.02 for every 5 years since cessation) There was an interaction between time since cessation and cumulative years of employment (*P* = 0.01), so that the effect decreased more among baggage handlers with 10 years or more of employment than among baggage handlers with less than 10 years of employment.

## Discussion

This large cohort study of 69,175 men examined the influence of occupational lifting in terms of cumulative years of employment as baggage handler on the risk of hospital admission because of incident lumbar disc herniation and LBP. We used register data on employment and diagnoses, so that exposure and outcome were measured independently and without any self-reported information. We found that baggage handlers had an increased incidence of LBP compared to the reference group. In addition, the IRR of LBP increased with cumulative years suggesting a dose–response relationship with duration of employment. There was a statistically significant interaction between age and baggage handler status, indicating that LBP which required treatment at hospital occurred at a younger age among baggage handlers than in the reference group. For baggage handlers working on the apron, the incidence was particularly increased in the period before introduction of extendable belt loaders. The incidence of LBP decreased when the baggage handlers terminated their employment. The baggage handlers did not have an increased incidence of lumbar disc herniation and this disorder was not associated with cumulative years as a baggage handler.

### Exposure

We were not able to assign exact exposure data in terms of intensity or frequency of lifting for each individual baggage handler. As exposure, we used job titles and number of years employed as a baggage handler which is a relatively crude measurement of exposure and this could bias our results towards null. Even though the main task for a baggage handler is manual handling of luggage and the exposure thus is rather homogeneous, we know that the amount of lifted luggage differs between the baggage handlers. However, we had very detailed information from the employers about the manually loaded baggage on the flights during many years. Based on these data, we have estimated an average exposure of lifting or handling of a total of 5000 kg a day, composed of baggage with an average mass of about 15 kg, which corresponds to lifting more than 300 times per day. Our reference group consisted of unskilled men to obtain socioeconomic comparability between the two groups. Some persons in the reference group may have had some heavy lifting at work, but altogether we believe that we have a significant contrast in occupational lifting between the two groups as only a few persons in the reference group would lift about 5000 kg per day. Even if our choice of referents may have reduced the contrast of exposure, this does not influence the effects of cumulative years of exposure within the group of baggage handlers.

A risk of misclassification of exposure is possible. We used several sources to define the employment periods, because employees from two different handling companies were included in the cohort of baggage handlers and information from the trade union supplemented data. The data were not intended to be used for research and were of different quality especially back in time before the registers became electronic. The local union member files became electronic starting in the 1980s and the company registers became electronic in 1990 and 1995. (Moller et al. 2017), so cumulative years of employment are probably not complete. However, a good agreement was found between the different sources, but a small proportion of persons who were registered as baggage handlers replied in the questionnaire that they had never worked as such (Mikkelsen et al. 2016). While a small misclassification in data exists, this is most likely non-differential.

A healthy worker effect could also have biased our results if men with recurrent back problems or sciatica are less likely to seek employment as a baggage handler or if more baggage handlers than referents with back problems left their job.

### Outcome

Our outcomes were based on hospital admissions and outpatient visits because of lumbar disc herniation and LBP registered in the Danish National Patient Register. The register covers the whole population, it is considered to be of high quality and the follow-up is almost complete (Lynge et al. 2011; Schmidt et al. 2015). Even if the register is of high quality, our outcome measure does not reflect the true occurrence of low back disorders and associated disability as only a small proportion of persons with low back pain are referred to hospital for investigation or treatment. Denmark has a public and free health care system, but it is also possible to consult private hospitals and clinics which may be a potential source of underreporting too. Although it has been mandatory since 2003 also for private health care providers to register all activities, registration from private hospitals and clinics is still incomplete (Schmidt et al. 2015). The proportion of treatments in private hospitals is very small though compared to the public health care system (Danish Health and Medicines Authority 2011). Use of register data also poses a risk of misclassification of outcome because of potential differences in registration practice across hospital departments, medical doctors, and calendar time. However, we consider that most clinicians will reliably distinguish between our two diagnostic groups, and we made sensitivity analyses restricted to diagnoses after 1994 and surgery after 1996 because of changes in the classification of diseases and surgery. This did not affect our results.

A possibility of referral bias exists if persons with low back pain are more likely to be referred to hospital when they have high physical work load, because their work is made difficult by their symptoms (Baker et al. 2003). Such differential referral would tend to inflate the risk estimates. However, our reference group consisted of men with manual work, and even though their work was not as heavy as baggage handling, most men in the reference group would also have problems with attending to their work with local low back pain. Furthermore, our results showed an internal dose–response relationship of LBP with cumulative years of exposure within the group of baggage handlers. It is not likely that referral bias would be associated with cumulative years.

In our study, we found no increased risk for lumbar disc herniation, but only for LBP among the baggage handlers. A possible explanation could be different etiopathogeneses of lumbar disc herniation and LBP. A few studies have used objective measures of low back disorders. Some studies have assessed lumbar disc degeneration in relation to occupation and back injuries using magnetic resonance imaging (Battie et al. 2008; Elfering et al. 2002; Luoma et al. 1998; Mariconda et al. 2007; Videman et al. 2007). These studies suggest that occupational exposures influence disc degeneration, but that disc degeneration in great part is determined by genetic factors. In a case–control study, Seidler et al. investigated the relationship between physical workload and symptomatic lumbar disc herniation with and without concomitant osteochondrosis or spondylosis (Seidler et al. 2003). They found only a statistically significant relation between cumulative exposure to lifting and carrying among cases with lumbar disc herniation when it was combined with osteochondrosis or spondylosis. They hypothesize that cumulative physical workload might only be related to spondylotic changes. This could indicate different etiopathogeneses of LBP and lumbar disc herniation, which again could be an explanation for our results only finding an association with LBP.

### Confounders

As we used register data, we did not have information on potential individual confounders such as BMI and lifestyle factors on all participants and could accordingly not adjust for these covariates in the analyses. Height, weight, and BMI and lifestyle factors as physical inactivity may influence back problems, especially among the baggage handlers who work in confined space in the aircraft compartments. However, we chose a reference group with similar socioeconomic status and made a questionnaire survey among the baggage handlers and a random sample of the reference group, and found that the two groups were comparable about lifestyle factors (Bern et al. 2013). In addition, we have adjusted for educational level. Considering these factors, we find it unlikely that our results are due to residual confounding.

### Strengths and limitations

The primary strength of the study is the design. The study was a longitudinal cohort study with a long follow-up period and hardly any loss to follow-up. The cohort was large and well defined, the information on exposure was recorded before the outcome and based on objective data, and data sources of information about exposure and outcome were independent. The main limitations are the lack of personal measurements of exposure, potential misclassification of exposure and outcome, and the outcome including only persons with severe enough back problems to be referred to hospital.

## Concluding remarks

Our study showed that occupational lifting among baggage handlers was a predictor of hospital admission due to LBP, but not lumbar disc herniation. The incidence of hospital admission or outpatient visit because of LBP was higher among baggage handlers than among referents. In addition, the incidence increased with cumulative years of work as a baggage handler and decreased after cessation of work as a baggage handler. For baggage handlers on the apron, the incidence was particularly increased in the period before introducing extendable belt loaders, suggesting that preventive measures to reduce cumulative work load may have had an effect.

## Appendix

See Table 4.

**Table 4:** Codes for diagnoses and surgical procedures used for lumbar disc herniation and low back pain

	ICD-8 codes for diagnoses	ICD-10 codes for diagnoses	Old codes for surgical procedures	NOMESCO codes for surgical procedures^a^
Lumbar disc herniation	72510, 72511	M51.0, M51.0A, M51.1, M51.1A, M51.1B, M51.1C, M51.1D, M51.1E, M51.1F, M51.1I, M51.2, M51.2A, M51.2B, M51.2C, M51.2D, M51.2E, M51.2F	77480, 82073, 82173	ABC16, ABC26
Low back pain	71709, 72879	M54.3, M54.4, M54.5, S33.5A		

^a^NOMESCO Classification of Surgical procedures (NCSP), version 1.16

## References

[CR1] Airaksinen O, Brox JI, Cedraschi C, Hildebrandt J, Klaber-Moffett J, Kovacs F, Mannion AF, Reis S, Staal JB, Ursin H, Zanoli G (2006). Chapter 4. European guidelines for the management of chronic nonspecific low back pain. Eur Spine J.

[CR2] Baker P, Reading I, Cooper C, Coggon D (2003). Knee disorders in the general population and their relation to occupation. Occup Environ Med.

[CR3] Barrero LH, Katz JN, Dennerlein JT (2009). Validity of self-reported mechanical demands for occupational epidemiologic research of musculoskeletal disorders. Scand J Work Environ Health.

[CR4] Battie MC, Videman T, Levalahti E, Gill K, Kaprio J (2008). Genetic and environmental effects on disc degeneration by phenotype and spinal level: a multivariate twin study. Spine (Phila Pa 1976).

[CR5] Battie MC, Videman T, Kaprio J, Gibbons LE, Gill K, Manninen H, Saarela J, Peltonen L (2009). The Twin Spine Study: contributions to a changing view of disc degeneration. Spine J.

[CR6] Bergsten EL, Mathiassen SE, Vingard E (2015). Psychosocial work factors and musculoskeletal pain: a cross-sectional study among Swedish flight baggage handlers. Biomed Res Int.

[CR7] Bern SH, Brauer C, Moller KL, Koblauch H, Thygesen LC, Simonsen EB, Alkjaer T, Bonde JP, Mikkelsen S (2013). Baggage handler seniority and musculoskeletal symptoms: is heavy lifting in awkward positions associated with the risk of pain?. BMJ Open.

[CR8] Bernard BP, Bernard B (1997). Musculoskeletal disorders and workplace factors, a critical review. Centers for disease control and prevention.

[CR9] Coenen P, Gouttebarge V, van der Burght AS, van Dieen JH, Frings-Dresen MH, van der Beek AJ, Burdorf A (2014). The effect of lifting during work on low back pain: a health impact assessment based on a meta-analysis. Occup Environ Med.

[CR10] Danish Health and Medicines Authority (2011) Private hospital activity 2006–2010 [In Danish: Aktivitet på private sygehuse 2006–2010]. Sundhedsstyrelsen

[CR11] De Beeck OR, Hermans V (2000). Research on work-related low back disorders.

[CR12] Elfering A, Semmer N, Birkhofer D, Zanetti M, Hodler J, Boos N (2002). Risk factors for lumbar disc degeneration: a 5-year prospective MRI study in asymptomatic individuals. Spine (Phila Pa 1976).

[CR13] Gallagher S (2005). Physical limitations and musculoskeletal complaints associated with work in unusual or restricted postures: a literature review. J Saf Res.

[CR14] Greenland S, Pearl J, Robins JM (1999). Causal diagrams for epidemiologic research. Epidemiology.

[CR15] Griffith LE, Shannon HS, Wells RP, Walter SD, Cole DC, Cote P, Frank J, Hogg-Johnson S, Langlois LE (2012). Individual participant data meta-analysis of mechanical workplace risk factors and low back pain. Am J Public Health.

[CR16] Hansson T (2001) [Low back pain and work]. Arbete och Hälsa 12:19–70. **(Swedish)**

[CR17] Harms-Ringdahl K, Hansson SO, Hagg O, Lundberg U, Mathiassen SE, Sundelin G, Svartengren M, Tropp H, Oberg B (2014). The importance of the working environment for back problems. A systematic review of literature.

[CR18] Heneweer H, Staes F, Aufdemkampe G, van Rijn M, Vanhees L (2011). Physical activity and low back pain: a systematic review of recent literature. Eur Spine J.

[CR19] Hoogendoorn WE, van Poppel MN, Bongers PM, Koes BW, Bouter LM (2000). Systematic review of psychosocial factors at work and private life as risk factors for back pain. Spine (Phila Pa 1976).

[CR20] Hoy D, Brooks P, Blyth F, Buchbinder R (2010). The Epidemiology of low back pain. Best Pract Res Clin Rheumatol.

[CR21] Kaila-Kangas L, Kivimaki M, Riihimaki H, Luukkonen R, Kirjonen J, Leino-Arjas P (2004). Psychosocial factors at work as predictors of hospitalization for back disorders: a 28-year follow-up of industrial employees. Spine (Phila Pa 1976).

[CR22] Koblauch H (2016). Low back load in airport baggage handlers. Dan Med J.

[CR23] Lenderink AF, Zoer I, Van Der Molen HF, Spreeuwers D, Frings-Dresen MH, van Dijk FJ (2012). Review on the validity of self-report to assess work-related diseases. Int Arch Occup Environ Health.

[CR24] Lotters F, Burdorf A, Kuiper J, Miedema H (2003). Model for the work-relatedness of low-back pain. Scand J Work Environ Health.

[CR25] Luoma K, Riihimaki H, Raininko R, Luukkonen R, Lamminen A, Viikari-Juntura E (1998). Lumbar disc degeneration in relation to occupation. Scand J Work Environ Health.

[CR26] Lynge E, Sandegaard JL, Rebolj M (2011). The Danish National Patient Register. Scand J Public Health.

[CR27] Mariconda M, Galasso O, Imbimbo L, Lotti G, Milano C (2007). Relationship between alterations of the lumbar spine, visualized with magnetic resonance imaging, and occupational variables. Eur Spine J.

[CR28] Martin BI, Deyo RA, Mirza SK, Turner JA, Comstock BA, Hollingworth W, Sullivan SD (2008). Expenditures and health status among adults with back and neck problems. JAMA.

[CR29] Martirosyan NL, Patel AA, Carotenuto A, Kalani MY, Belykh E, Walker CT, Preul MC, Theodore N (2016). Genetic alterations in intervertebral disc disease. Front Surg.

[CR30] Mikkelsen S, Brauer C, Pedersen EB, Alkjaer T, Koblauch H, Simonsen EB, Helweg-Larsen K, Thygesen LC (2016). A cohort study on meniscal lesions among airport baggage handlers. PLoS One.

[CR31] Moller KL, Brauer C, Mikkelsen S, Loft S, Simonsen EB, Koblauch H, Bern SH, Alkjaer T, Hertel O, Becker T, Larsen KH, Bonde JP, Thygesen LC (2017). Copenhagen Airport Cohort: air pollution, manual baggage handling and health. BMJ Open.

[CR32] Nordic Medico-Statistical Committee (2011) NOMESCO Classification of Surgical Procedures (NCSP), version 1.16. Nordic Medico-Statistical Committee (NOMESCO)

[CR33] Pedersen CB (2011). The Danish civil registration system. Scand J Public Health.

[CR34] Pincus T, Burton AK, Vogel S, Field AP (2002). A systematic review of psychological factors as predictors of chronicity/disability in prospective cohorts of low back pain. Spine (Phila Pa 1976).

[CR35] Plouvier S, Leclerc A, Chastang JF, Bonenfant S, Goldberg M (2009). Socioeconomic position and low-back pain–the role of biomechanical strains and psychosocial work factors in the GAZEL cohort. Scand J Work Environ Health.

[CR36] Schmidt M, Schmidt SA, Sandegaard JL, Ehrenstein V, Pedersen L, Sorensen HT (2015). The Danish National Patient Registry: a review of content, data quality, and research potential. Clin Epidemiol.

[CR37] Seidler A, Bolm-Audorff U, Siol T, Henkel N, Fuchs C, Schug H, Leheta F, Marquardt G, Schmitt E, Ulrich PT, Beck W, Missalla A, Elsner G (2003). Occupational risk factors for symptomatic lumbar disc herniation; a case-control study. Occup Environ Med.

[CR38] Shiri R, Falah-Hassani K, Heliovaara M, Solovieva S, Amiri S, Lallukka T, Burdorf A, Husgafvel-Pursiainen K, Viikari-Juntura E (2019). Risk factors for low back pain: a population-based longitudinal study. Arthritis Care Res (Hoboken).

[CR39] Shrier I, Platt RW (2008). Reducing bias through directed acyclic graphs. BMC Med Res Methodol.

[CR40] Stalhammar HR, Leskinen TP, Kuorinka IA, Gautreau MH, Troup JD (1986). Postural, epidemiological and biomechanical analysis of luggage handling in an aircraft luggage compartment. Appl Ergon.

[CR41] Statista—statistics and market data about transport and logistics (2019). https://www.statista.com/statistics/193533/growth-of-global-air-traffic-passenger-demand/. Accessed 14 June 2019

[CR42] Stock SR, Fernandes R, Delisle A, Vezina N (2005). Reproducibility and validity of workers’ self-reports of physical work demands. Scand J Work Environ Health.

[CR43] Tafazzol A, Aref S, Mardani M, Haddad O, Parnianpour M (2016). Epidemiological and biomechanical evaluation of airline baggage handling. Int J Occup Saf Ergon.

[CR44] The Danish Data Protection Agency (2008) Standard terms for research projects—the act on processing of personal data

[CR45] The International Air Transport Association (IATA) (2019). https://www.iata.org/publications/store/Pages/20-year-passenger-forecast.aspx. Accessed 14 June 2019

[CR46] The National Committee on Health Research Ethics (DNVK) Copenhagen (2011) Guidelines about notification etc. of a biomedical research project to the committee system on biomedical research ethics

[CR47] Thygesen LC, Daasnes C, Thaulow I, Bronnum-Hansen H (2011). Introduction to Danish (nationwide) registers on health and social issues: structure, access, legislation, and archiving. Scand J Public Health.

[CR48] Thygesen LC, Mikkelsen S, Pedersen EB, Moller KL, Alkjaer T, Koblauch H, Simonsen EB, Moller SP, Brauer C (2016). Subacromial shoulder disorders among baggage handlers: an observational cohort study. Int Arch Occup Environ Health.

[CR49] Undeutsch K, Gartner KH, Luopajarvi T, Kupper R, Karvonen MJ, Lowenthal I, Rutenfranz J (1982). Back complaints and findings in transport workers performing physically heavy work. Scand J Work Environ Health.

[CR50] Videman T, Levalahti E, Battie MC (2007). The effects of anthropometrics, lifting strength, and physical activities in disc degeneration. Spine (Phila Pa 1976).

[CR51] Waddell G, Burton AK (2001). Occupational health guidelines for the management of low back pain at work: evidence review. Occup Med.

[CR52] Wahlstrom J, Burstrom L, Nilsson T, Jarvholm B (2012). Risk factors for hospitalization due to lumbar disc disease. Spine (Phila Pa 1976).

[CR53] Wahlstrom J, Burstrom L, Johnson PW, Nilsson T, Jarvholm B (2018). Exposure to whole-body vibration and hospitalization due to lumbar disc herniation. Int Arch Occup Environ Health.

